# Seroepidemiology of infectious bovine rhinotracheitis infection in unvaccinated cattle

**DOI:** 10.14202/vetworld.2015.1416-1419

**Published:** 2015-12-18

**Authors:** M. Saravanajayam, K. Kumanan, A. Balasubramaniam

**Affiliations:** 1 Veterinary University Training and Research Centre, Perambalur - 621 220, Tamil Nadu, India; 2 Tamil Nadu Veterinary and Animal Sciences University, Chennai - 600 051, Tamil Nadu, India

**Keywords:** cattle, enzyme-linked immunosorbent assay, infectious bovine rhinotracheitis, prevalence

## Abstract

**Aim:**

The present study aimed to investigate the seroepidemiology of infectious bovine rhinotracheitis (IBR) infection in the non-vaccinated cattle population in northern part of Tamil Nadu, India.

**Materials and Methods:**

A total of 255 sera samples were collected from cattle having the history of respiratory and reproductive disorder from cattle of different age, breeds, and sex. All the sera samples were subjected to indirect ELISA for the diagnosis of IBR antibodies.

**Results:**

Results revealed that the seroprevalence of IBR infection among non-vaccinated cattle population was of 65.88%. No significant difference was noticed in the prevalence of IBR infection between cattle showing respiratory (63.64%) and reproductive form (70.89%) (p≥0.05). A higher prevalence was noticed in animals above 3 years of age (59.60%) and in crossbred animals (71.26%) than young and non-descript animals. This study showed the higher prevalence of IBR infection in female (67.92%) than in male (33.33%).

**Conclusion:**

Cattle population in this part can better be protected with vaccination than leaving them unvaccinated and sero-monitoring shall have to be stressed with regular attempts to isolate and characterize the causative agent for IBR.

## Introduction

Infectious bovine rhinotracheitis (IBR) caused by bovine herpesvirus Type 1 (BHV1) is a common cattle disease which is responsible for the significant huge economic loss in dairy industry worldwide. Infection with BHV-1 is associated with mild to severe respiratory disease and represents a high-risk for bovine respiratory disease complex. Infections with this agent can also manifest as ocular, neonatal, gastrointestinal, and neurologic disease as well as reproductive failure due to abortion and other genital symptoms (infectious pustularvulvovaginitis (IPV) and infectious pustularbalanoposthitis (IPB) [1,2]. Though the disease is essentially a herd problem, occurs mostly in animals over 6 months of age. Transmission occurs normally by contact with infected animals, aerosol route and virus-contaminated semen from BHV-1 infected bulls. A complication associated with IBR infection is the ability of the virus to establish latency unless stress conditions favor its reactivation [3].

Indirect enzyme-linked immunosorbent assay (ELISA) has been extensively used for the assessment of seroepidemiological investigation of IBR antibodies among the cattle population in the various parts of the world [1,4-7].

In the current study, the intensive management system of cattle population without IBR vaccination and frequent movement of animals between different places were the factors taken into the account to study the prevalence of IBR infection. Hence, the present study analyzed the prevalence of IBR infection among cattle population in organized farms as well as cattle brought to the Madras Veterinary College Hospital and Saidapet Polyclinic, Chennai and relationship of disease prevalence with respect to age, breed and sex were assessed simultaneously. Since, seroepidemiology of IBR was carried out the 1^st^ time in this region, the paired sera sampling and consideration of latent carriers were not done.

## Materials and Methods

### Ethical approval

All the protocols were as per the Committee for the Purpose of Control and Supervision of Experiments on Animals guidelines and approved by the Institutional Animal Ethics Committee of Madras Veterinary College.

### Place of work

The study was conducted at the Department of Veterinary Epidemiology and Preventive Medicine with the help of Department of Animal Biotechnology, Madras Veterinary College, Tamil Nadu Veterinary and Animal Sciences University, Chennai, Tamil Nadu, India.

### Antigen and known sera samples

The BHV-1 strain BFA-OCS-W was passaged in Madin-Darby bovine kidney (MDBK) cell line used as a standard virus for antigen preparation. OIE international standard sera for IBR that include IBR-EU1 serum (strong positive) and IBR-EU3 serum (negative) from bovine origin received from Central Institute for Animal Disease Control Lelystad, The Netherlands, were utilized for this study as an internal control.

### Sera samples

A total of 255 cattle sera samples for which the details are given in Tables-1-4 were drawn from the organized farms as well as cattle brought to Madras Veterinary College Hospital and Saidapet Polyclinic, Chennai with the history of respiratory and reproductive disorders and the samples were considered as representative for the cattle population in the study area.

**Table-1:** Prevalence of IBR antibodies with respect to history of respiratory and reproductive infection in cattle.

Type of infection	No. of sera tested	No. of positives	Percentage of positives
Respiratory infection	176	112	63.64*
Reproductive infection	79	56	70.89*

* No significant difference between respiratory and reproductive form (p≥0.05), IBR=Infectious bovine rhinotracheitis

**Table-2:** Age wise prevalence of IBR antibodies in cattle.

Age (year)	No. of sera tested	No. of positives	Percentage of positives
Below 1	18	5	27.78*
13	21	11	52.38
36	104	71	68.27*
Above 6	112	81	72.32**

* Significant difference noticed between the age groups of below 1 year and 3-6 years, below 1 year and above 6 years (p<0.01), IBR=Infectious bovine rhinotracheitis

**Table-3:** Breed wise prevalence of IBR antibodies in cattle.

Breed	No. of sera tested	No. of positives	Percentage of positives	Overall percentage
Crossbred				
Jersey Crossbred	127	90	70.87	71.26*
Holstein Friesian Crossbred	47	34	72.34	
Native breeds				
Murrah	34	18	52.94	54.32*
Surti	20	12	60.00	
Non-descript	27	14	51.85	

* The prevalence rates are significantly higher in crossbred animals than indigenous breeds (p<0.01), IBR=Infectious bovine rhinotracheitis

**Table-4:** Sex wise prevalence of IBR antibodies in cattle.

Sex	No. of sera tested	No. of positives	Percentage of positives
Male	15	5	33.33*
Female	240	163	67.92*

* The prevalence rates are significantly higher in female animals than male (p<0.01), IBR=Infectious bovine rhinotracheitis

### Titration of the BHV1

About 50 µl of MDBK cell suspension with 50 µl of growth medium were dispersed into the 96 well tissue culture plates. After confluent monolayer formation, the growth medium was removed and inoculated with 50 µl of tenfold dilution of BHV-1 using three wells per log dilution of virus. The plate was kept at 37°C for 1 h for adsorption of the virus into the cells and then the virus inoculum was removed and added with 100 µl of maintenance medium to each well. The plate was read for the degree of CPE and TCID_50_ was calculated 7 days post-infection [8]. Confirmation of virus was made by hematoxylin and eosin staining of BHV-1 infected MDBK coverslips.

### ELISA

ELISA is a sensitive and specific test for assessing the antibodies for any diseases. Moreover, ELISA is prescribed a test for detecting IBR antibodies as per OIE.

### Preparation of antigen

BHV-1 infected monolayer with tissue culture fluid in Roux flask was harvested by freezing and thawing thrice. Then, the fluid was centrifuged at 5000 × g for 10 min at 4°C to remove the cell debris. The supernatant fluid was sonicated for 5 times (1 min at a time with an interval of 1 min each) under ice pack with an ultrasonic disintegrator (BransarSonifier 450).

Again the tissue culture fluid was centrifuged at 5000 × g for 30 min at 4°C. The supernatant was collected in ultracentrifuge tubes and centrifuged at 1,00,000 × g for 1 h at 4°C. The resulting pellet was resuspended to 1/5^th^ volume of phosphate buffer saline (pH 7.2) and stored at –20°C. A control antigen was prepared in a similar manner from non-infected MDBK cells. The estimation of protein concentration was done for both the test and control antigen [9].

The optimum concentrations of coating antigen, sera dilution, antibovineIg G-HRP conjugate were standardized by the checkerboard titration as 1:50, 1:10 and 1:50, respectively. Indirect ELISA [10] was performed with slight modification.

The mean optical density (OD) value which was twice and above the negative OD value was taken as positive. The control positive and negative sera used were OIE IBR reference sera.

### Statistical analysis

The data obtained from this study were statistically analyzed using Student’s *t*-test [11].

## Results

### Propagation and titration of BHV-1

The final titer of 10^6.362^ TCID_50_ per 50 µl of the BHV-1 was obtained after four serial passages in MDBK cell line which was used for performing the indirect ELISA in this study. Characteristic CPE was observed in the cell line 2 days post-infection with BHV-1.

### ELISA

The protein concentration of BHV-1 tissue culture antigen was found 0.6 g per 100 ml [9]. The mean OD value of known negative serum (IBR-IU3) was 0.080, twice (0.160), and above this OD value was taken as positive.

Among 255 sera samples were screened from cases of cattle brought to the clinics and cattle in organized farms, 168 samples (65.88%) were found to be positive for IBR antibodies by indirect ELISA. The results are shown in Table-5.

**Table-5:** Prevalence of IBR antibodies in cases attended clinics and Dairy Cattle in organized farms.

Name of the place	No. of sera tested	No. of positives	Percentage of positives
Madras Veterinary College Hospital, Chennai- 7.	142	97	68.31
Saidapet Polyclinic, Chennai	12	8	66.67
Livestock Research Station, Kattupakkam	75	48	64.00
Private Dairy Farm, Chennai District	12	8	66.67
Private Diary Farm, Thiruvallur District	14	7	50.00
Total	255*	168	65.88

* No significant difference in the prevalence of IBR antibody among the serum samples collected from different sources (p≥0.05), IBR=Infectious bovine rhinotracheitis

### IBR antibody prevalence in the animals with history of suspected signs

Albeit there are reports in other parts of the country, this is the first report about the prevalence of IBR antibodies in this region. The prevalence of IBR antibodies based on the history of respiratory and reproductive disorders were 63.64% and 70.89%, respectively, (Table-1). Analysis of data revealed that no significant difference in prevalence of IBR antibodies existed between the prevalence of respiratory and reproductive disorders (p≥0.05).

### Age wise prevalence

In the present study, the differences in IBR prevalence rate between below 1 year and 1-3 years groups was not significant, whereas between below 1 year and 3-6 years and above 6 years groups were highly significant (p<0.01) and the results were depicted in Table-2.

### Breed wise prevalence

The breed wise prevalence of IBR antibodies were 71.26% and 54.32% in crossbred cows (Jersey Crossbred and Holstein Friesian Crossbred) and native buffalo breeds (Murrah, Surti and non-descriptive), respectively, (Table-3). The prevalence was significantly higher in crossbred animals than indigenous breeds (p<0.01).

### Sex wise prevalence

The prevalence of IBR antibodies in male and female that differed significantly (p<0.01) were 33.33% and 67.92%, respectively, as shown in Table-4.

## Discussion

The prevalence of IBR antibodies among cattle population reared in part of south India was confirmed. As per the earlier reports in India, 56.5% positive reactors in various parts of India and also 64.7% positives in Andhra Pradesh were recorded [12,13]. IBR infection was noticed with high prevalence among cattle reared as large herd [14].

### IBR antibody prevalence in the animals with history of suspected signs

The higher prevalence in reproductive disorder was probably due to natural service by infected bulls and artificial insemination with infected semen [1,2,15]. The respiratory form of prevalence was due to the frequent introduction of cattle from various parts of the country and intensive management practices of cattle [16,17].

### Age wise prevalence

The age wise prevalence to IBR infection denoted that there was an increasing trend in the occurrence of IBR infection as age advances, and prevalence was low in the young animals [5,18,19]. In the present study, it is revealed that the prevalence of IBR in animals over 3 years of age was found to be higher than the lower age groups.

### Breed wise prevalence

All breeds are susceptible [20] and the native breeds were comparatively less susceptible which is possibly due to their inherent resistance to infection.

### Sex wise prevalence

The high prevalence in female might be attributed to the use of infected bulls for natural service and infected semen for artificial insemination [15,21-23].

## Conclusion

The current study on the seroepidemiology of IBR infection revealed that unvaccinated cattle in northern part of Tamil Nadu were exposed to BHV-1. In conclusion, the cattle population in this part can better be protected with vaccination than leaving them unvaccinated and sero-monitoring shall have to be stressed with regular attempts to isolate and characterize the causative agent for IBR to minimize the economic loss in dairy farming arising out of IBR.

## Authors’ Contributions

MS planned the study design, collected and examined the samples. KK carried out the cell culture work and AB drafted and revised the manuscript. All authors read and approved the final manuscript.
